# VR Sickness Adaptation With Ramped Optic Flow Transfers From Abstract To Realistic Environments

**DOI:** 10.3389/frvir.2022.848001

**Published:** 2022-05-23

**Authors:** Isayas Adhanom, Savannah Halow, Eelke Folmer, Paul MacNeilage

**Affiliations:** 1 Department of Computer Science and Engineering, University of Nevada, Reno, NV, United States; 2 Department of Psychology, University of Nevada, Reno, NV, United States

**Keywords:** virtual reality, VR sickness, simulator sickness, optic flow, cybersickness, adaptation

## Abstract

VR sickness is a major concern for many users as VR continues its expansion towards widespread everyday use. VR sickness is thought to arise, at least in part, due to the user’s intolerance of conflict between the visually simulated self-motion and actual physical movement. Many mitigation strategies involve consistently modifying the visual stimulus to reduce its impact on the user, but this individualized approach can have drawbacks in terms of complexity of implementation and non-uniformity of user experience. This study presents a novel alternative approach that involves training the user to better tolerate the adverse stimulus by tapping into natural adaptive perceptual mechanisms. In this study, we recruited users with limited VR experience that reported susceptibility to VR sickness. Baseline sickness was measured as participants navigated a rich and naturalistic visual environment. Then, on successive days, participants were exposed to optic flow in a more abstract visual environment, and strength of the optic flow was successively increased by increasing the visual contrast of the scene, because strength of optic flow and the resulting vection are thought to be major causes of VR sickness. Sickness measures decreased on successive days, indicating that adaptation was successful. On the final day, participants were again exposed to the rich and naturalistic visual environment, and the adaptation was maintained, demonstrating that it is possible for adaptation to transfer from more abstract to richer and more naturalistic environments. These results demonstrate that gradual adaptation to increasing optic flow strength in well-controlled, abstract environments allows users to gradually reduce their susceptibility to sickness, thereby increasing VR accessibility for those prone to sickness.

## 1 INTRODUCTION

Virtual reality (VR) is currently seen as the next digital Frontier. In recent years, various commercial VR head-mounted displays (HMD) have become available at a low cost and VR is already widely used for training, rehabilitation, education, work, and entertainment. Unfortunately, VR sickness is still considered a major barrier to mass consumer adoption (Nelson, 2015). VR sickness is a type of motion sickness unique to VR and can include symptoms such as nausea, pallor, sweating, stomach awareness, increased heart rate, drowsiness, disorientation, and general discomfort (Kennedy et al., 1993). Not all individuals who are exposed to VR will experience VR sickness, however, those who do may find it debilitating (Regan, 1995; Stanney et al., 2003).

Several VR sickness mitigation strategies have already been developed, including the use of dynamic or foveated field-of-view (FOV) restriction (Fernandes and Feiner, 2016; Adhanom et al., 2020; Yamamura et al., 2020) and vection blurring (Budhiraja et al., 2017). In practice though, the bulk of locomotor VR experiences that are currently available rely primarily on the use of teleportation (Al Zayer et al., 2020; Monteiro et al., 2021) which has been shown in a recent meta review (Prithul et al., 2021) to result in significantly lower VR sickness incidence but at the cost of lower presence and potential spatial disorientation. The reason for the success of teleportation may be in part due to the lack of optic flow generated during use (Prithul et al., 2021). Optic flow can contribute to both vection (Palmisano et al., 2011; Seno et al., 2018) and sensory conflict (Bubka et al., 2007; Palmisano et al., 2011), which cause sickness in certain contexts (Bubka et al., 2007).

A current challenge with VR sickness is that available mitigation strategies aren’t 100% effective since the effectiveness of any particular mitigation strategy varies across individuals (Reason, 1978). This makes it difficult to create a one-size-fits-all strategy for mitigation. A better strategy may be to supplement existing mitigation techniques by investigating how the user might best prepare themselves for continued VR use.

Research which focuses on using adaptation to the virtual environment has shown promising results for VR sickness mitigation (Reason and Brand, 1975; Cobb et al., 1999; Hill and Howarth, 2000; Bailenson and Yee, 2006; Domeyer et al., 2013; Beadle et al., 2021). The general assumption is that this repeated exposure facilitates adaptation to sensory conflict in the virtual environment, reducing sickness. However, much of this success is dependent on the researchers exposing users to the *same* virtual environment. Development of a training paradigm which facilitates the transfer of adaptation effects between multiple environments has prove more difficult to achieve (Mouloua et al., 2005; Smither et al., 2008; Duzmanska et al., 2018). These issues may be in part a result of the dissimilarities between the environments (Reason and Brand, 1975; Reason, 1978); adaptation to sensory conflict is thought to be largely dependent on the user becoming accustomed to the conflict which arises in a particular environment through developing the appropriate expectations for sensory input in that environment (Reason and Brand, 1975; Reason, 1978; Keshavarz, 2016). Therefore, facilitating more general adaptation may require training through exposure to factors that are consistent between all VR environments, for example, optic flow stimuli.

Therefore, we aimed to develop a training technique that facilitates the transfer of adaptation effects across environments. Our study borrows concepts from motion sickness mitigation work by emphasizing gradual exposure to sickness-inducing stimuli (Rine et al., 1999; Takeda et al., 2001) such as expanding optic flow (Ebenholtz, 1992; Bubka et al., 2007) and graphic realism (Golding et al., 2012; Davis et al., 2015) to facilitate adaptation. To this end, we use a simplified training environment consisting only of the walls and floor of a labyrinth that users navigate. Over successive days, we gradually adapt users to increasing optic flow by incrementally increasing luminance contrast of the visual stimulus. We show that the adaptation that is achieved using this simplified environment with gradual increases in optic flow strength successfully transfers to a richer, more naturalistic environment. Results of this study suggest that it should be possible to develop standardized training paradigms to reduce and possibly even eliminate VR sickness in susceptible individuals.

## 2 RELATED WORK

There are three main theories aimed at explaining VR sickness: postural instability theory, eye movement theory, and sensory conflict theory (Reason and Brand, 1975; Riccio and Stoffregen, 1991; Ebenholtz, 1992). Postural instability theory (Riccio and Stoffregen, 1991) attributes VR sickness to a disruption of postural stability caused by unnatural or unexpected motion in the virtual environment. Eye movement theory (Ebenholtz, 1992) postulates that optic flow drives rapid reflexive eye movement to stabilize the image (optokinetic nystagmus) and that the associated sensory and motor signals innervate the vagal nerve, leading to VR sickness. Sensory conflict theory (Reason and Brand, 1975) seems to be the most widely accepted (Kolasinski, 1995; Keshavarz et al., 2014) and attributes VR sickness to the conflicting signals between the visual, vestibular, and somatosensory senses causing discomfort and nausea among other symptoms. These theories are not mutually exclusive, and this list does not encompass all efforts to explain VR sickness (Keshavarz et al., 2015).

One element these theories have in common is that they all seek to explain how sickness may be caused by exposure to full-field visual motion, or optic flow. Optic flow is thought to cause VR sickness (Davis et al., 2015; Tian et al., 2022) particularly when the visually-simulated self-motion does not match natural self-motion of the user, i.e. when there is sensory conflict (Bubka et al., 2007). Many mitigation strategies, including FOV restriction, aim to reduce optic flow during movement (Fernandes and Feiner, 2016; Adhanom et al., 2020; Shi et al., 2021). Other strategies instead try to reduce conflict by providing some form of vestibular or efferent feedback (Peng et al., 2020), for example, by walking-in-place (Tregillus and Folmer, 2016). It is important to note that optic flow is not the same as vection; optic flow refers to the visual motion stimulus itself while vection refers to the visually-induced perception of self-motion (Fujii and Seno, 2020). Optic flow can cause vection (Palmisano et al., 2015; Seno et al., 2018), so their separate effects can be easily confounded during discussions of VR sickness (Chang et al., 2020). Like optic flow, vection has been linked to feelings of VR sickness (Keshavarz et al., 2015; Palmisano et al., 2017), however the relationship is still not perfectly understood (Keshavarz et al., 2015; Palmisano et al., 2017) nor should it be oversimplified. As vection may be experienced without inducing VR sickness, vection alone is not sufficient to induce sickness (Keshavarz et al., 2015). Instead, it must be paired with other factors such as sensory conflict, postural instability, etc. This makes it difficult to disentangle the distinct effects of the optic flow versus vection when investigating sickness.

Other work related to the current study has investigated adaptation to VR sickness. Prior studies have argued that repeated exposure to an aversive stimulus allows developing an altered expectation of sensory input (Reason and Brand, 1975; Keshavarz, 2016), with the required length of exposure increasing as the stimuli become more complex (Reason, 1978). It has been suggested that this makes it unlikely for adaptation effects in one virtual environment to transfer to another. The degree of transfer may depend on *the degree of dissimilarity* between two environments (Reason and Brand, 1975; Reason, 1978). Previous research in similar environments has been rather consistent in demonstrating effective adaptation, with reports of symptoms decreasing over the course of exposure (Hill and Howarth, 2000; Bailenson and Yee, 2006; Howarth and Hodder, 2008; Domeyer et al., 2013; Beadle et al., 2021), even on timescales as low as 45 min (Sinitski et al., 2018). In one study by Lampton et al. (2000), the researchers saw VR sickness decrease following repeated exposure to a training environment, however, these effects disappeared when the participants entered the mission environment (Lampton et al., 2000). Another study by Hill and Howarth (2000) had participants view a game being played through an HMD on five consecutive days. Results also demonstrated significantly less nausea on the fifth day, compared to the first. More recently, a study by Beadle et al. (2021) had participants perform a shooting task in an HMD over 3 sessions. SSQ scores significantly decreased following each session. This is not an exhaustive list of VR adaptation studies (for review, we recommend Duzmanska et al. (2018)), but they do demonstrate a common theme; adaptation to a single VR environment is possible for mitigating VR sickness. In this experiment, we hope to expand upon this research by examining whether adaptation can transfer between VR environments.

Other relevant prior work has examined how various methods of training against sickness may offer a potential solution to VR sickness more generally, rather than to a single environment (Mouloua et al., 2005; Smither et al., 2008). One such study by Smither et al. (2008) showed that when participants were pre-exposed to simulated rotary stimulation they experienced less symptoms of VR sickness than individuals who had no prior exposure (Smither et al., 2008). Similarly, an experiment by Mouloua et al. (2005) demonstrated cross-platform training was possible, but that it was only successful when using optokinetic nystagmus (OKN) training, and not a VR HMD (Mouloua et al., 2005). Another study by Pöhlmann and colleagues (2021) found that individuals that spend extensive amounts of time playing action video games (not on HMDs) experienced less VR sickness when exposed to a simple virtual environment compared to individuals that do not play any video games (Pöhlmann et al., 2021). All of these experiments were examining transfer in a way that used a non-VR device as their training mechanism. It is possible that these experiments were training against motion sickness more broadly, rather than adaptation to VR specifically. While this may be another effective methodology for sickness mitigation, it stands to reason that this would not be readily available for the average user. Here, we seek to facilitate adaptation solely through the use of a VR HMD.

Specifically, we explicitly aim to facilitate adaptation between distinct VR environments. This means that experiencing one environment will lead to a reduction in VR sickness in a dissimilar environment. Additionally, we hypothesize that this adaptation can occur without exposing participants to the full optic flow and luminance contrast normally experienced in the VR environment. In other words, training can be tailored to act as a “ramp up” period for the participants. Studies which center on training against motion sickness have suggested that adaptation should be facilitated through a gradual increase of the provocative stimulus (Rine et al., 1999; Takeda et al., 2001). Similarly, if we want to train participants against VR sickness, best practice may be to increase intensity over time such that adaptation has time to take place while avoiding extreme illness.

## 3 MATERIALS AND METHODS

Our study aims to answer two research questions:

- **RQ 1.** Does ramped exposure to increased optic flow strength in an abstract environment allow gradual adaptation?
- **RQ 2.** Does the adaptation transfer from abstract to more complex environments?

### 3.1 Equipment

We used an HTC Vive Pro Eye HMD to present stimuli, and track position and orientation. It has a diagonal FOV of 110°, a refresh rate of 90Hz, and a combined resolution of 2880 × 1600 pixels. It allows the user to adjust the interpupillary distance (IPD) and focal distance of the HMD to their comfort. The HMD was powered by an AMD Ryzen 7 1700x Eight-Core processor with NVIDIA GeForce GTX 1080ti graphics card and 16 GB of memory.

An Empatica E4 wristband was used to read Electrodermal Activity (EDA) from participants. We use the Empatica’s lead wire extension to collect data using Ag/AgCl electrodes attached to the palmar surface. Participants navigated the environment and provided discomfort score input using an XBox 360 controller. This controller is preferred over the Vive touchpad given its familiarity to new users. The tracking space was set to 2.2 by 2.4 m.

Our system included stimulus presentation and data acquisition components. Stimulus presentation was accomplished through a custom application developed in Unity3D engine version 2019.4.28, using SteamVR version 1.7 plugin. For data acquisition, we used lab streaming layer (LSL) along with LSL LabRecorder version v1.14.2. LSL allows unified and time-synchronized collection of various data streams in an experiment.

The Empatica E4 streaming server was used along with a custom built application that used PyLSL version 1.15.0 to stream the EDA data from the wristband to LSL LabRecorder. EDA data was collected at a sampling rate of 4 Hz.

### 3.2 Virtual Environment

To address our two research questions, we developed two separate virtual environments: an adaptation training environment and a test environment. The training environment was an abstract environment developed to help us answer RQ1, and the test environment was a natural and complex virtual environment developed to help us answer RQ2.

The training environment was a custom-built labyrinth. The labyrinth consists of a series of repeating hallways with alternating black and white stripes on the walls, and arrows on the floor to help guide participants to the waypoints. This environment was built to have fewer features than the city environment. Unlike the test environment, the training environment did not change in elevation, all paths were straight, sharp turns, and not winding, and the appearance stayed relatively constant throughout. Our hope with this environment was to reduce feature richness, as previous research has suggested that virtual environments with greater graphic realism (Golding et al., 2012; Davis et al., 2015) and scene complexity (Kingdon et al., 2001; Stanney et al., 2003) can lead to higher levels of sickness than simple or abstract scenes. Thus, we created an abstract environment that allowed us to maintain and control optic flow strength through manipulation of luminance contrast. Luminance contrast is the ratio between the maximum and minimum luminance values in the environment, and it is calculated using the following formula (Sauvan and Bonnet, 1993; Patterson and York, 2009; Guo et al., 2021):

(1) $$C\;=\;\frac{\left(I_{max\;}-I_{min}\right)}{\left(I_{max\;}+I_{min}\right)}$$

With *I*_max_ and *I*_min_ representing the highest and lowest luminance. In our training environment, luminance contrast was set to a value of 0.50 during the first day, 0.75 the second, and 1.0 (full contrast) on the final day. Increasing luminance contrast leads to increased optic flow strength and vection (Guo et al., 2021), and thus serves as a method to gradually increase stimulus intensity over successive training days.

The test environment was adapted from the Windridge City (UnityTechnologies, 2021) environment. This environment was chosen due to its rich features; it consists of a city surrounded by lush forests, flower fields, and winding dirt roads. This test environment replicates what users may experience in normal VR use, particularly for entertainment. The environment has compelling imagery, changes in elevation, and provides plenty of interesting features for participants to examine as they navigate it. For these reasons, the test environment also works as a direct comparison for real-world applications. Both environments contained a set of waypoints as their main means of navigating the environments for 20 min.

**FIGURE 1 |:**
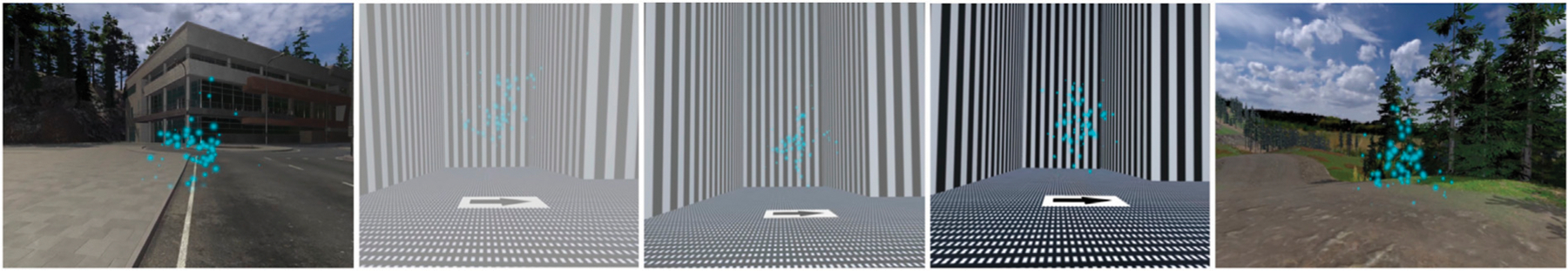
Participants were exposed to two virtual environments over the course of 5 days. On days 1 and 5, participants navigated a naturalistic environment, which served as the baseline and test for adaptation. On days 2 through 4, participants navigated an optokinetic labyrinth with an increase in the luminance contrast on each day. Participants used blue particle cloud waypoints and arrow textures to help guide their navigation.

In summary, the training and testing environments were very dissimilar. This ensures that any reduction in sickness on the final test day is not simply due to repeated exposure to the same environment. Instead, we examine whether adaptation acquired in the training environment is maintained and transfers to the test environment on the final day. Figure 1 shows the baseline and final testing environment used on days 1 and 5 on the left and right, and the training environment with increasing contrast luminance levels used on days 2 through 4 in the center.

### 3.3 Measurements

Measuring VR sickness has proven challenging and best practices are still being debated. Most commonly, researchers have relied on the Simulator Sickness Questionnaire (Kennedy et al., 1993) but updated versions such as the VR Sickness Questionnaire (Kim et al., 2018) have recently gained some traction. The SSQ measures multiple symptoms using a long questionnaire and is usually collected post exposure. More recently, researchers have developed more simple alternative questionnaires that can be collected during the experiment. The Discomfort Score (Fernandes and Feiner, 2016; Adhanom et al., 2020) is a widely used method which asks subjects to rate how sick they are feeling at that moment on a scale of 0–10 during the VR experience, with 0 indicating how they felt when the experience started, and 10 indicating that they need to stop usage immediately. This metric has an obvious advantage over the previously mentioned questionnaires; the Discomfort Score allows us to see sickness as it evolves in users over time.

Subjective measurements of VR sickness such as the SSQ and Discomfort scores have some limitations because they rely on the user’s subjective judgment which could vary between individuals. In an effort to establish more objective measures of tracking VR sickness, researchers have looked to collect physiological data such as postural sway (Häkkinen et al., 2002), electrodermal activity (EDA) (Magaki and Vallance, 2019), body temperature (Geršak et al., 2020), and heart rate (Yamamura et al., 2020). Eye blink data has also been suggested as a potential source of valuable sickness information (Chang et al., 2021). In this study, to measure the incidence of VR sickness and discomfort we use both subjective and objective methods.

#### 3.3.1 Subjective Sickness Scores

##### 3.3.1.1 Discomfort Scores

The discomfort scores were collected through the VE by prompting the user to select a score every minute. The discomfort score allows a sampling of VR sickness scores during the trial. Discomfort scores were averaged for each participant per session to obtain an average discomfort score (ADS), and an ending discomfort score (EDS) was calculated by using the last discomfort score for each participant per session, similar to Fernandes and Feiner (2016). This value is 10 if the participant terminated early due to severe discomfort.

##### 3.3.1.2 SSQ Scores

Baseline SSQ data was collected before each session and post-immersion SSQ data was collected after each session. Data collected from the SSQ questionnaires are used to calculate four associated scores, namely: Total Severity, Oculomotor, Nausea, and Disorientation scores. These scores were calculated as per the conversion formulas by Kennedy et al. (1993). We then subtract the baseline SSQ subscores from the post-immersion SSQ subscores to get our relative SSQ subscores.

#### 3.3.2 Objective Sickness Scores

**FIGURE 2 |:**
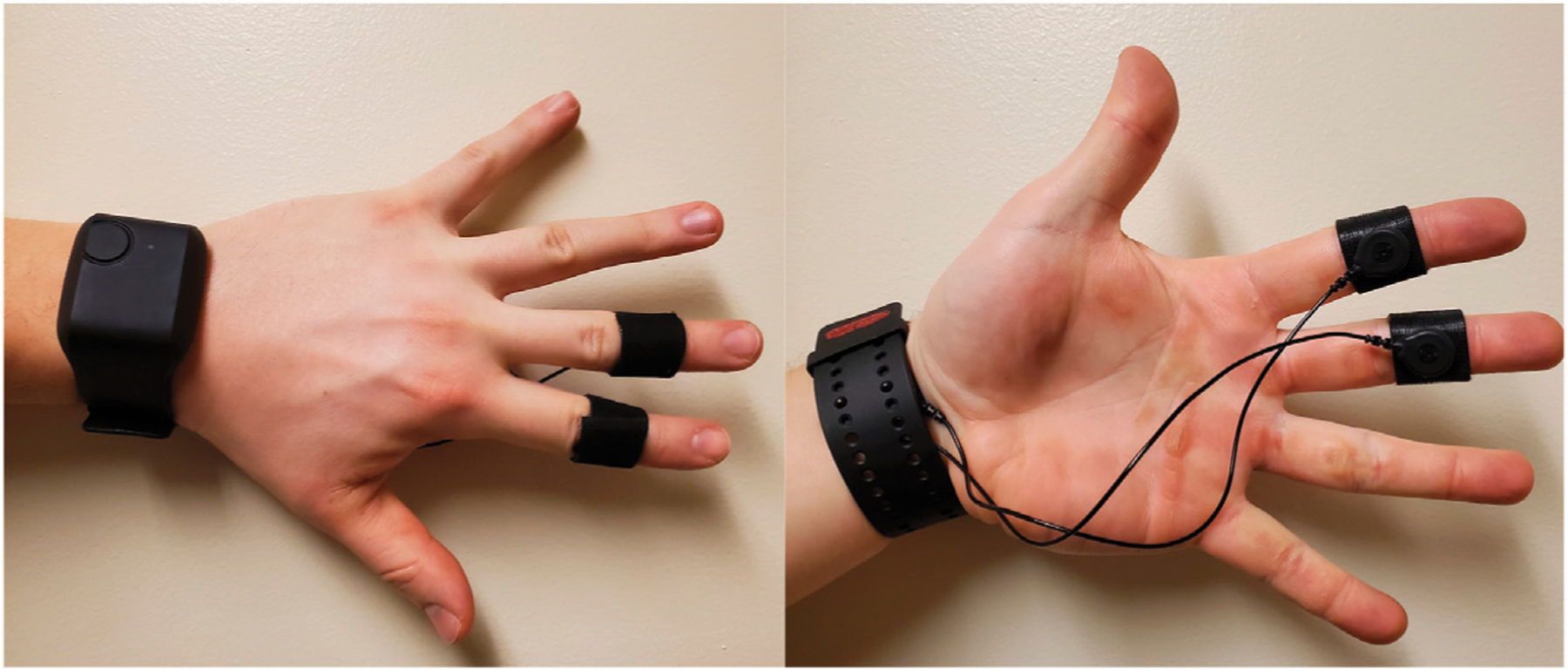
Placement of the Empatica E4 wristband and the EDA electrodes.

A recent review of the causes and measurements of VR sickness by Chang et al. (2020) found that electrodermal activity (EDA) is one of the widely used objective measures of VR sickness. As a result, in this study we chose to use EDA as an objective measurement of VR sickness. EDA was recorded from the medial-phalanges of the index and middle finger of the non-dominant hand’s palmar sites. That is, we placed the sensors on the upper-middle portion of the index and middle finger (see Figure 2). Baseline EDA data was recorded for 2 min before immersion in VR, and for the full period of each experiment session. There are two main components in EDA signals: the general tonic-level component measured as Skin Conductance Level (SCL) which is thought to reflect general changes in autonomic arousal, and the phasic-level component measures as Skin Conductance Response (SCR) which refers to the fast changing elements of the signal.

To decompose the EDA signal into the phasic and tonic components we applied a high pass filter with a cut-off frequency of 0.05 Hz, and we compute the SCL of the tonic component and Amplitude Root Mean Square (RMS) from the phasic component as these metrics have been shown to be promising VR sickness indicators in previous studies (Dennison et al., 2016; Gavgani et al., 2017). Similar to previous studies (Dennison et al., 2016; Gavgani et al., 2017) we divide the recorded data into blocks of 1 min each, and for the purpose of statistical analysis the tonic SCL and the RMS amplitude are averaged for these blocks.

To remove individual variability, our tonic SCL scores are standardized by dividing all exposure values by the baseline score (Braithwaite et al., 2013; Dennison et al., 2016), and our EDA signal was standardized using Z-score standardization prior to extracting the phasic component (Braithwaite et al., 2013).

#### 3.3.3 Optic Flow

To understand the amount of optic flow experienced by participants during each day of the experiment, we measured the magnitude of optic flow during each session. To measure optic flow we recorded video streams of participants navigating the virtual environment during each condition. Similar to previous studies, the video streams were recorded from the left stereoscopic camera (Islam et al., 2021). The streams were recorded at a rate of 30 frames per second and at the full resolution of 2220 × 1450. The video frames were preprocessed by re-scaling them to 392 × 256 resolution, as calculating dense optic flow with the full resolution would be computationally expensive. We use the dense optic flow calculation algorithm developed by Farneb (2003) to calculate the magnitude and direction of optic flow in each frame. Optic flow for each frame is expressed as a 2D vector field where each vector represents displacement of image points between successive frames. We then average the magnitude of optical flow, measured as pixels per frame, over all pixels of each frame to get the average magnitude of optic flow per frame. The data for all frames of each session video is then averaged to get the average magnitude of optic flow exposure for that session. For convenience, we refer to this average magnitude below as optic flow strength.

### 3.4 Procedure

When participants arrived for the first day of testing, they were provided information explaining the procedures of the experiment, the risks involved, and the types and handling procedures of the data collected in the experiment. Following this, participants were asked to fill out a pre-study demographic questionnaire. The participants were shown a 2-min video explaining the virtual environments and the experiment task and were given an overview of the controller used in the study and how to use it.

Participants started by filling in a baseline SSQ. They were then assisted with putting the physiological sensor wristband on their non-dominant hand. Following this, 2 minutes of resting baseline EDA data outside the VR environment was collected from the participants. Participants were asked to sit and avoid movement of the hand wearing the wristband during baseline data collection. Following this, participants were assisted with putting on the VR headset. We made sure the HMD and wristband were well-fitted, but comfortable.

Participants were asked to navigate the Windridge City environment by following a set of waypoints to the end of the environment. On days 2 through 4, participants navigated an optokinetic labyrinth at increasing luminance contrast values: 0.5 on day 2, 0.75 on day 3, full contrast on day 4. On the final day of testing, participants navigated the Windridge city environment as they had on the first day. During the experiment, participants were also asked to provide a discomfort score (see Section 3.3.1) (Fernandes and Feiner, 2016) at 1 minute intervals. Participants completed the navigation tasks while standing. They controlled their forward and lateral movements with the XBox 360 controller and steered with their head. To prevent transfer of symptoms between sessions, a minimum of 12 h (maximum 48 h) and a full night’s rest were required between sessions. Each experiment session took around 30 min, with 20 min of VR exposure.

After each session, participants were asked to fill out a Simulator Sickness Questionnaire (SSQ (Kennedy et al., 1993)) in order to assess their level of post-exposure sickness. The room was kept at 20°C to prevent interference with SSQ data (i.e. sweating due to temperature, rather than sickness) and ensure participants all experience the same air temperature (Arnold et al., 2019).

### 3.5 Participants

Potential participants were recruited by online flyers from the local campus community. Participants who expressed interest in the study were given a questionnaire to determine their eligibility. The questionnaire (Supplementary Figure S1) was adapted from Golding (2006) and Kinsella (2018). Because we were interested in users who experience VR sickness symptoms, participants were excluded from participating if they satisfied any of the following conditions: frequent users of VR, never experience VR sickness or motion sickness, suffer from inner ear problems or vertigo. Initially, 49 people showed interest in the study of whom 12 were excluded because they had frequent experience with VR. Two were excluded because they have inner ear problems or vertigo. 13 chose not to participate in the study after getting contacted for participation. 22 participants were recruited for the experiment (11 male, 11 female, mean age: 23.45, and SD: 3.9).

Out of the 22 participants two participants, both female, were unable to complete the study reporting that they were too sick to continue after day 2. One additional female participant experienced data loss during a session due to equipment failure. The analyses included were conducted on the remaining 19 participants (11 male, 8 female. mean age: 22.8, SD: 3.6). The number of participants was chosen based on similar previous studies (Al Zayer et al., 2016; Fernandes and Feiner, 2016; Adhanom et al., 2020).

## 4 RESULTS

To align our results with our research questions, we report the results in two parts: the first subsection reports results for the test sessions (days 1 and 5) and the second subsection reports the results for the training sessions (days 2, 3, and 4). The results for the test sessions aim to address RQ2 - whether the adaptation training was transferred from the abstract training environment to the complex testing environment.

For all statistical tests, we used Shapiro–Wilk tests to test the normality of our data, and we used the box plot method to identify outliers outside the Q1 − 1.5 × IQR to Q3 + 1.5 × IQR range. Q1 and Q3 are the first and third quartile, respectively. IQR is the interquartile range (IQR = Q3 – Q1). There were outliers in some of our results due to participants showing high levels of discomfort, especially during the early days. We retained these outliers because they are a natural part of the data, rather than errors. A significance level of 0.05 was set for all analysis. All our statistical tests were performed in R version 4.1.0 using the rstatix package, and results were duplicated SPSS.

### 4.1 Testing Sessions

In this section we report the results for day 1 (pre-training) and day 5 (post-training). We report summary results of the discomfort scores and the SSQ subscales in Table 1 and we discuss the results and our statistical tests in more detail in the following subsections.

#### 4.1.1 Discomfort Scores

**FIGURE 3 |:**
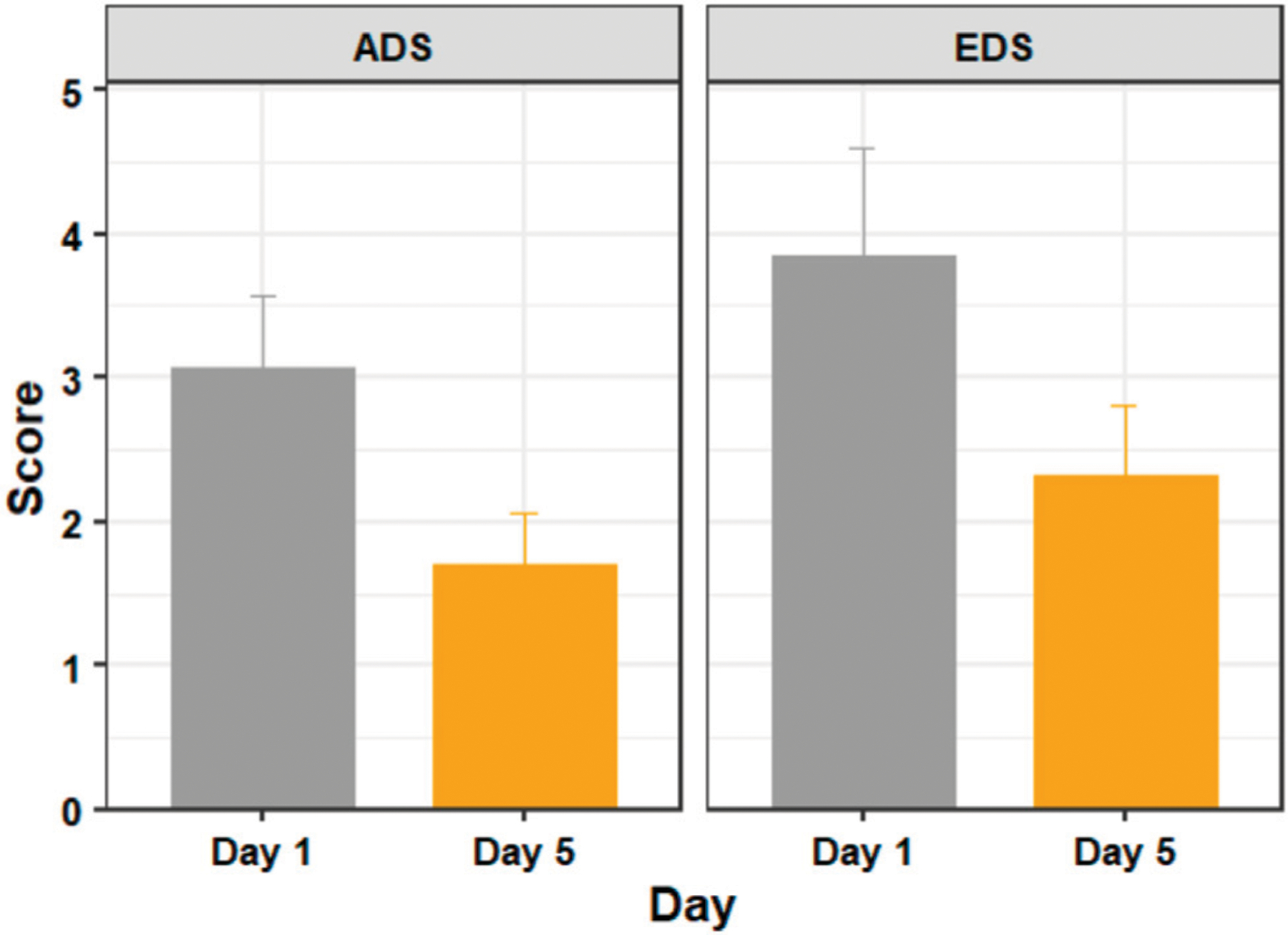
The average discomfort score (ADS) and ending discomfort score (EDS) of participants on days 1 (before training) and 5 (after training). Figure shows mean and standard error.

Figure 3 shows the mean ADS and EDS scores of the testing days 1 and 5. A Shapiro–Wilk test found that our data were normally distributed. Our outlier test showed that we had one outlier, but no extreme outliers. We retained the identified outlier in each score, as we considered that they were a natural part of the data, rather than errors.

Using a paired t-test we found significant differences in discomfort measures between days 1 and 5 with respect to both ADS (*t* (18) = 3.297, *p* = 0.004, *d* = 0.756) and EDS (*t* (18) = 2.635, *p* = 0.017, *d* = 0.605).

#### 4.1.2 SSQ Sub-scores

**FIGURE 4 |:**
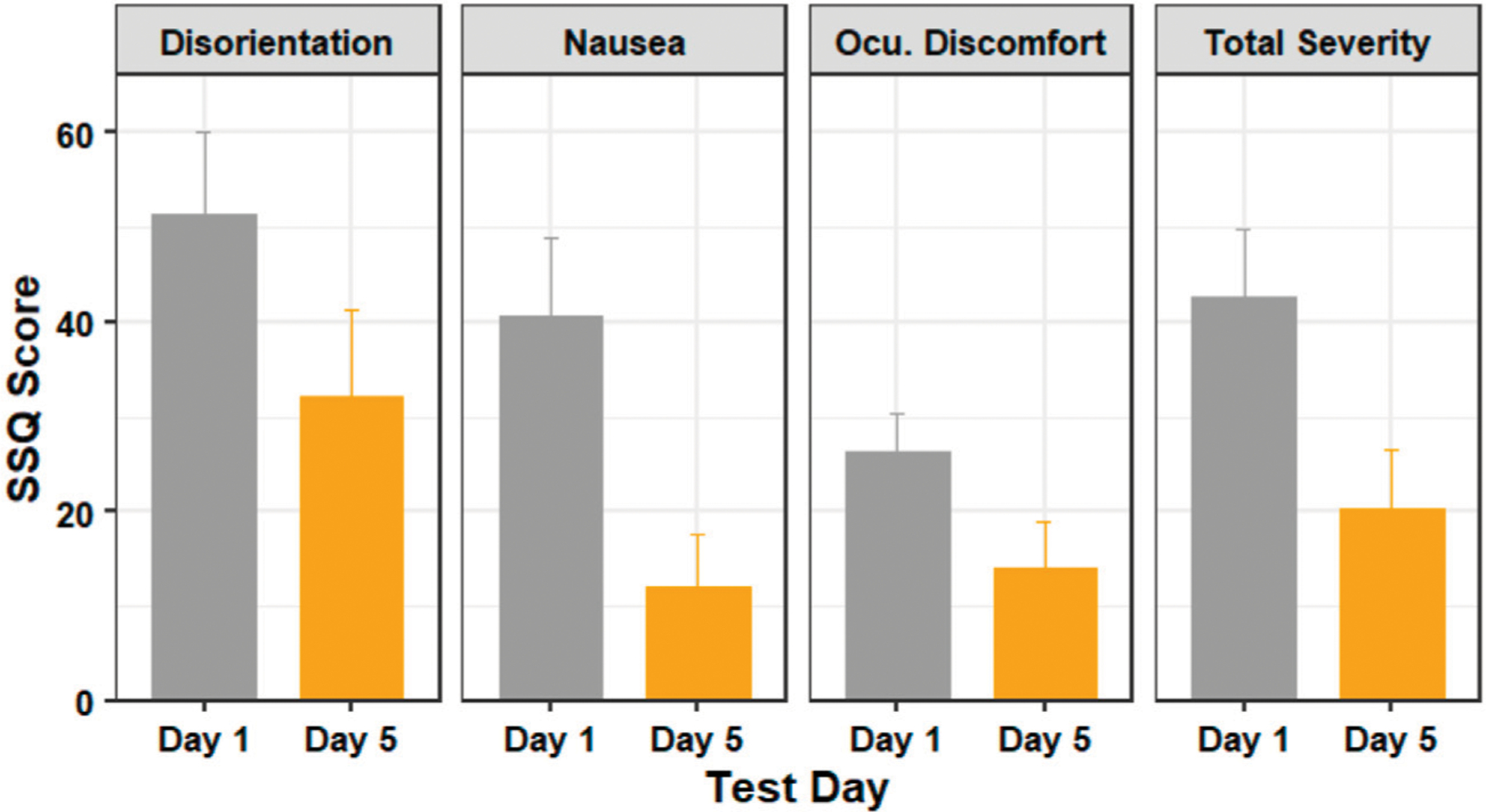
The SSQ subscores of participants in days 1 (before training) and 5 (after training). Figure shows mean and standard error.

Figure 4 shows the average SSQ subscores for the testing days. A Shapiro–Wilk test found that our data were not normally distributed. Our outlier test showed that we had some outliers. We retained the identified outliers in each sub-score, as these scores were from users who felt severe discomfort during the experiment and were not extreme outliers. Since our data violates the assumptions of the paired t-test, we used a Wilcoxon signed-rank test - the non-parametric alternative to the paired t-test.

A Wilcoxon signed-rank test showed that there was statistically significant change in nausea score (*Z* = 16, *p* = 0.001) and the total severity score (*Z* = 14, *p* = 0.031), but there was no statistically significant change in the disorientation score (*Z* = 12, *p* = 0.143) or the oculomotor discomfort score (*Z* = 13, *p* = 0.096) between days 1 and 5.

#### 4.1.3 EDA Data

A Wilcoxon signed rank test showed that, on day 1, the phasic amplitude RMS values did not differ significantly when compared to the baseline values (*Z* = 115, *p* = 0.322). On day 5, there was no significant increase in phasic amplitude RMS values during the session (*Z* = 174, *p* = 0.0.322). Comparing between days, while there were no significant differences at the baseline between days 1 and 5 (*Z* = 146, *p* = 0.973), the phasic amplitude RMS was significantly lower on day 5 compared to day 1 (*Z* = 215, *p* = 0.015) indicating a decrease of VR sickness symptoms in day 5, after completing the training sessions.

**FIGURE 5 |:**
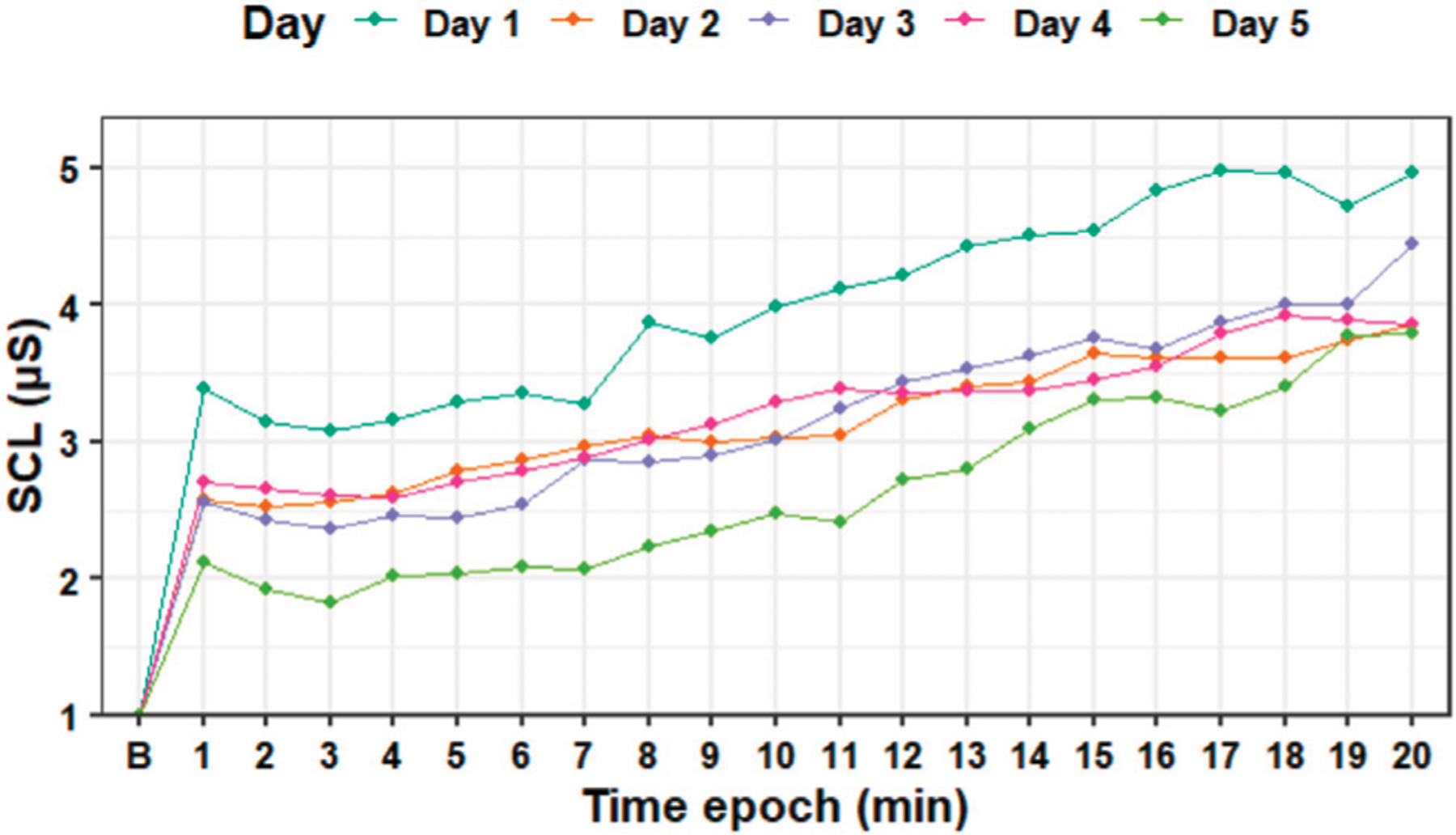
Standardized tonic skin conductance level data averaged for each epoch (minute) during the experiment days. B—baseline SCL.

Figure 5 shows how the average tonic SCL values changed over time during each session. Although the SCL plot shows a decrease on the tonic SCL level between days 1 and 5, a Wilcoxon signed-rank test showed that the difference was not significant (*Z* = 18, *p* = 0.648).

#### 4.1.4 Optic Flow Magnitude

A Shapiro–Wilk test found that our optic flow magnitude data for the testing days were not normally distributed, therefore we used the Wilcoxon-signed ranks test to analyze the data.

Our analysis of optic flow magnitude data with the Wilcoxon-signed rank test for the testing days (days 1 and 5) found that participants did not experience a significantly different magnitude of optic flow (*Z* = 0.648, *p* = 0.517) during the test days.

### 4.2 Training Sessions

In this section we report our results for the training days (days 2, 3, and 4). We report summary results of the discomfort scores and the SSQ subscales in Table 2 and we discuss the results and our statistical tests in more detail in the following subsections.

#### 4.2.1 Discomfort Scores

**FIGURE 6 |:**
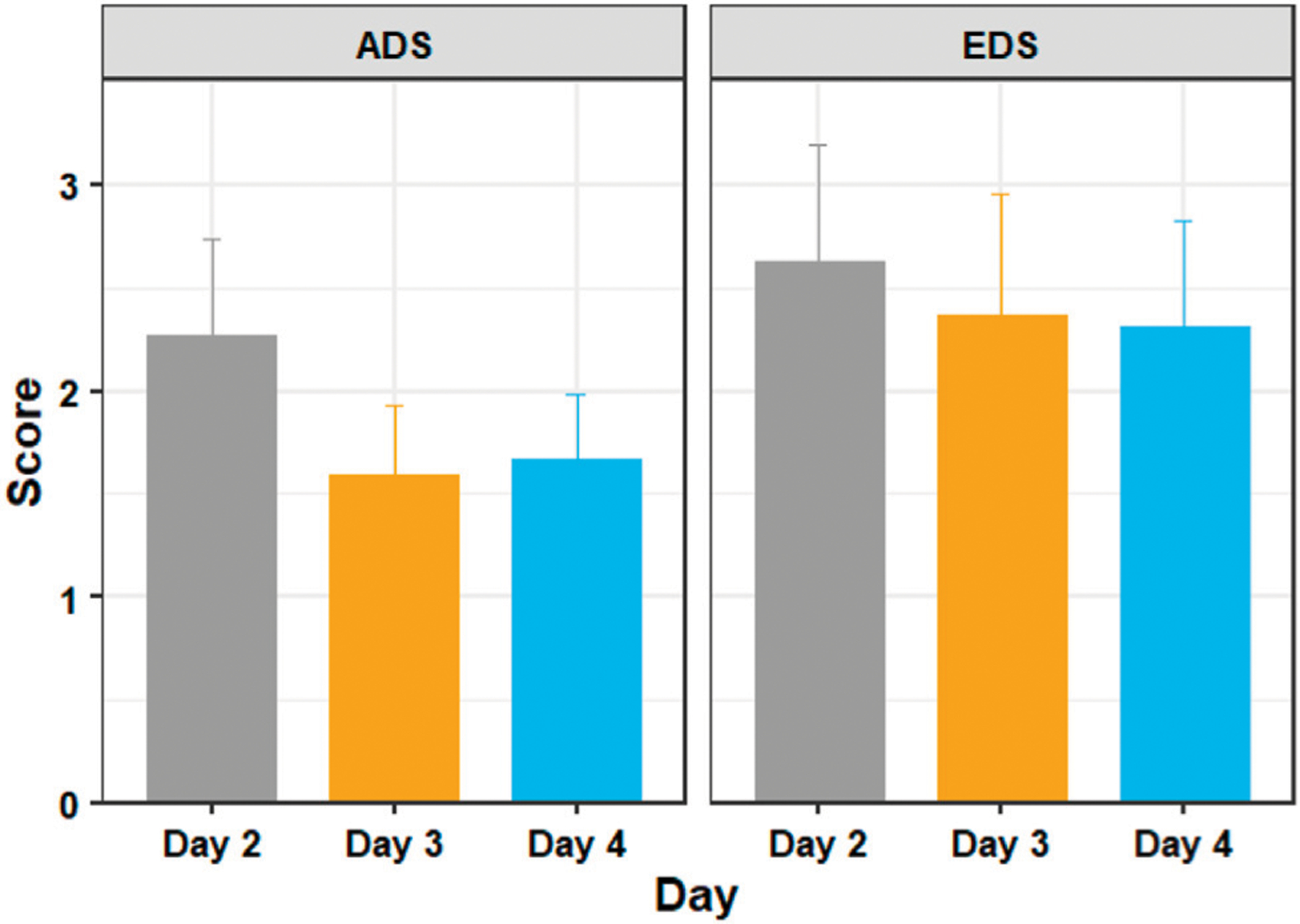
The average discomfort score (ADS) and ending discomfort score (EDS) of participants in days 2, 3, and 4 of adaptation training. Figure shows mean and standard error.

Figure 6 shows the mean ADS and EDS scores of the training days 2, 3, and 4. A Shapiro–Wilk test found that our data were normally distributed. We did not have extreme outliers in our data.

A one way repeated measures ANOVA showed that there were significant differences in discomfort measures between the training days with respect to ADS (*F* = 4.086, *p* = 0.042) but not with respect to EDS (*F* = 0.503, *p* = 0.609). Post hoc analysis with paired t-tests was conducted and Bonferroni adjustment applied. The post hoc tests revealed that ADS was statistically significantly decreased from day 2 to day 3 (*t* (18) = 3.38, *p* = 0.010), but not from days 3–4 (*t* (18) = −0.31, *p* = 1.000). EDS did not show any statistically significant decreases between day 2 and day 3 (*t* (18) = 0.96, *p* = 1.000) or from day 3 to day 4 (*t* (18) = 0.15, *p* = 1.000).

#### 4.2.2 SSQ Sub-Scores

**FIGURE 7 |:**
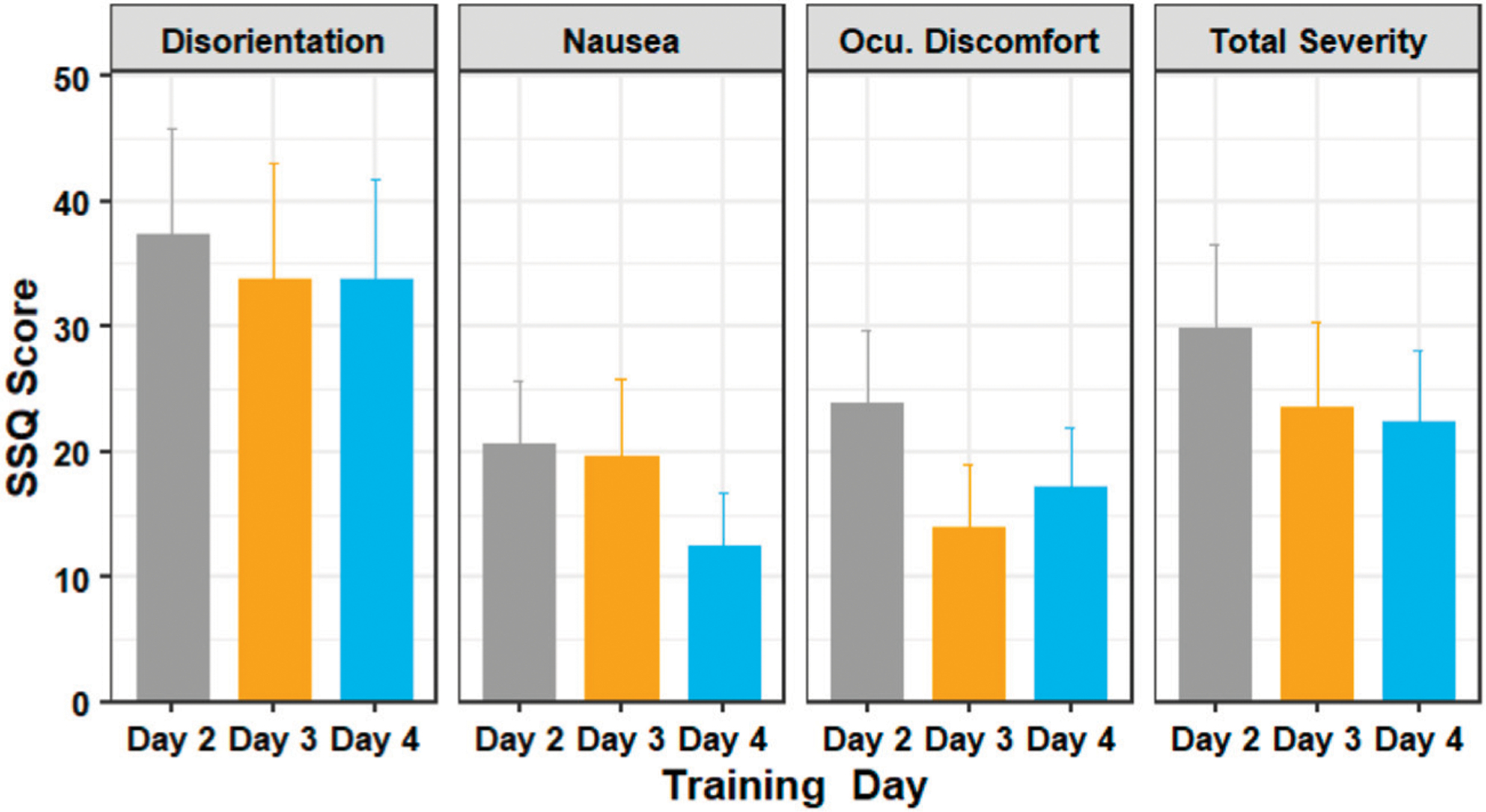
The SSQ subscores of participants in days 2 through 4 of the training session. Figure shows mean and standard error.

Figure 7 shows the average SSQ subscores for the training days. A Shapiro–Wilk test found that our data were not normally distributed. Our outlier test also showed that we had some outliers from users who felt extreme discomfort during the experiment. Since our data violates the assumptions of the one way repeated measures ANOVA, we used the Friedman test - the non-parametric alternative to the one way repeated measures ANOVA.

The Friedman test showed that there was a statistically significant difference in the oculomotor discomfort score among the training days, (*χ*^2^ (2) = 8.38, *p* = 0.015). The test showed that there was no significant difference in the nausea score (*χ*^2^ (2) = 2.76, *p* = 0.251), disorientation score (*χ*^2^ (2) = 2.52, *p* = 0.284) and the total severity score (*χ*^2^ (2) = 3.65, *p* = 0.161). Post-hoc analysis with Wilcoxon signed-rank tests was conducted with a Bonferroni correction applied, and showed that there was no significant difference in scores between the training days.

#### 4.2.3 EDA Data

Wilcoxon signed rank tests showed that the phasic amplitude RMS values did not increase significantly at the end of the session for days 2 (*Z* = 147. *p* = 0.945), 3 (*Z* = 145.5. *p* = 0.986) and 4 (*Z* = 109. *p* = 0.231) when compared to the baseline values. Comparing between days 2, 3, and 4, a Friedman test showed that there were no significant differences for the baseline (*χ*^2^ (2) = 2.471, *p* = 0.291) or the session end (*χ*^2^ (2) = 0.471, *p* = 0.790) values of the phasic amplitude RMS. A Friedman test showed that the tonic SCL data also does not show significant difference between days 2, 3, and 4 (*χ*^2^ (2) = 1.529, *p* = 0.465).

#### 4.2.4 Optic Flow Magnitude

**FIGURE 8 |:**
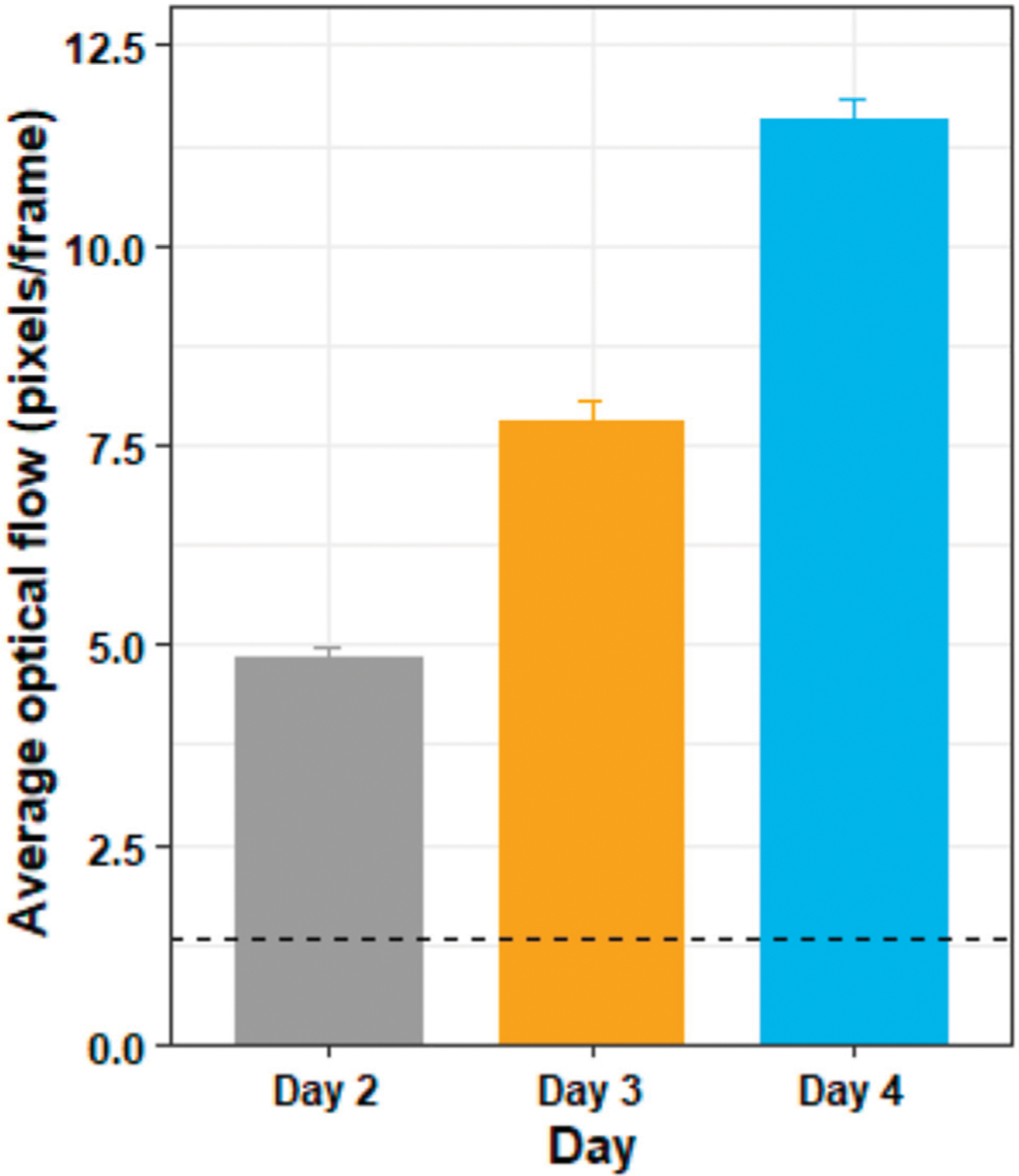
The average optic flow magnitude by day for the training days. The horizontal dotted line shows the average optic flow magnitude of the two testing days (days 1 and 5) for comparison. The figure shows mean and standard deviation.

**FIGURE 9 |:**
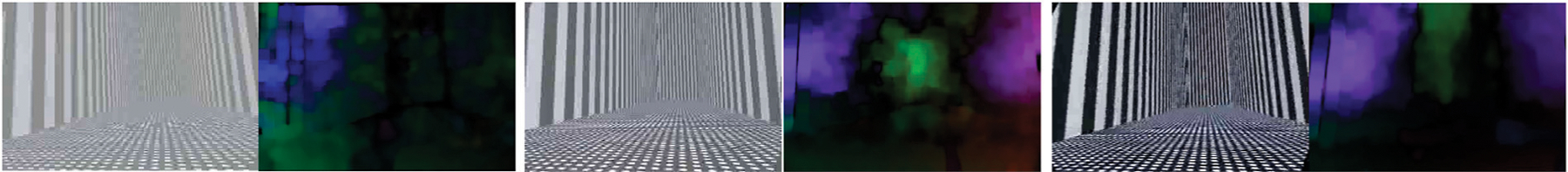
Frames captured in days 2, 3, and 4 of training days along with their corresponding optic flow visualization. The frames show the view a typical participant saw traveling through the same spot at the same speed on the three separate days of the experiment, along with their optic flow visualizations.

Figure 8 shows the average optic flow magnitude for the training days, and Figure 9 shows a visualization of the optic flow a typical user experienced during the training days. A Shapiro–Wilk test found that our data for both the training days were not normally distributed, therefore we used the Friedman test to analyze the results for the training days.

The Friedman test showed that there was a substantially significant difference in optic flow magnitude between the training days (*χ*^2^ (2) = 38, *p* < 0.05). Post hoc analysis with Wilcoxon signed-rank tests was conducted with a Bonferroni correction applied, and showed that there was significant difference in optic flow magnitude between days 2 and 3 (*Z* = 0, *p* < 0.05), between days 3 and 4 (*Z* = 0, *p* < 0.05) and between days 2 and 4 (*Z* = 0, *p* < 0.05).

## 5 DISCUSSION

This study had two aims, to investigate VR sickness adaptation methods that allow gradual exposure to increasing optic flow and the transferability of adaptation from one environment to another. To avoid floor effects, we explicitly recruited individuals that reported susceptibility to VR sickness and limited VR use. Subjects were tested over 5 days, consisting of a baseline on day 1, three consecutive training days, and a final test session on day 5. The test environment consisted of a cityscape, while the training environment was a less realistic labyrinth with black and white striped walls. Contrast and thus optic flow strength was gradually increased over consecutive training days. Results indicate that, despite the increase in optic flow strength, subjective sickness reports and objective physiological measures did not increase, but instead tended to remain constant or even decrease over consecutive training days, though this decrease was generally not significant. Comparison between measures obtained on days 1 and 5, on the other hand, tended to show significant decreases in sickness. These results suggest that exposure to ramped optic flow strength supports gradual adaptation of VR sickness and its symptoms. Additionally, adaptation that is developed in more abstract and impoverished environments can transfer to richer and more naturalistic environments. These results suggest that carefully controlled adaptation procedures can be a successful mitigation strategy for VR sickness.

### 5.1 RQ_1_: Ramped Optic Flow Exposure

One of the primary aims of this study was to investigate whether exposure to ramped optic flow strength in an abstract environment allows for gradual adaptation to VR sickness. Prior studies have shown that reducing overall optic flow strength, for example, by restricting the FOV to eliminate faster motion at the periphery (Fernandes and Feiner, 2016) can lead to reduced sickness. In addition, reducing contrast has recently been shown to reduce vection magnitude and duration, and to increase vection onset latency (Guo et al., 2021). However, to our knowledge, no prior studies have explicitly demonstrated that scene contrast directly impacts VR sickness. It is reasonable to hypothesize that decreased contrast, and thus decreased optic flow strength, should lead to decreased severity of sickness. For this reason, we chose to begin training on day 2 with a reduced contrast version of the training environment. The drop in reported sickness between days 1 and 2 suggests that the low-contrast labyrinth was indeed less provocative than the full-contrast test environment, and thus a well-chosen training stimulus. We believe this reduction is due in part to the impoverished nature of the training environment (Stanney et al., 2003; Riecke et al., 2006; Davis et al., 2015) and the reduced contrast, but it may also bea manifestation of the typical day-1-to-day-2 learning rate reported previously (Newman et al., 2013). Regardless, contrast manipulation provided a convenient method to parametrically increase optic flow strength (Figure 8), vection (Guo et al., 2021), and thus (presumably) the provocative nature of the VR experience, over successive training days. Despite this increase, we did not observe an increase in reported sickness. To the contrary, reported sickness either remained constant or decreased over successive training days. These results suggest that manipulating optic flow via contrast manipulation may be a straightforward way to maintain sickness at manageable levels while still allowing training.

### 5.2 RQ_2_: Adaptation Transfer

Another primary aim of this study was to investigate whether adaptation to VR sickness that was developed in a more abstract environment can transfer to a richer and more complex environment. Prior research has demonstrated that VR sickness is more pronounced with increasing graphic realism (Golding et al., 2012; Davis et al., 2015) and scene complexity (Kingdon et al., 2001; Stanney et al., 2003). This was our motivation for training using an impoverished environment, as a way to limit sickness during training. But for such a method to be effective, the results of any adaptation must be shown to transfer from more impoverished to richer environments. To our knowledge, no other study has demonstrated the transfer of adaptation between VR environments using VR as its training mechanism. We take the reduction in sickness observed on Day 5 relative to Day 1 in our experiment to be evidence that adaptation developed in the labyrinth environment effectively transferred to the richer test environment. An alternative explanation for the reduced sickness on day 5 relative to day 1 is that this is simply a manifestation of the reduction in sickness due to prior exposure to the test environment on day 1. We believe this is unlikely because the sickness scores on day 4 with the training stimulus (ADS, EDS, and SSQ) are generally very similar to the sickness scores observed on day 5 with the test stimulus. This similarity is consistent with the idea of adaptation transfer and seems unlikely to occur by chance. Not all subscores from the SSQ indicated decreased sickness, specifically, the oculomotor and disorientation scores were not significantly different between Day 1 and Day 5. This would indicate that the change in total severity score is driven mostly by a change in nausea. It is worth noting that research also suggests that an increased sense of presence can lead to a decrease in VR sickness (Weech et al., 2019). While we did not collect information on subjects’ feelings of presence, we take the drop in sickness between days 1 and 2 to demonstrate that regardless of presence, subjects did experience greater sickness in the original, complex, environment.

## 6 LIMITATIONS AND FUTURE WORK

The conclusions of the study would be strengthened by collection of additional data to control for both ramping optic flow as well as the transfer of adaptation between abstract and rich natural environments. For example, the drop in sickness and EDA observed between days 1 and 5 could be compared against the drop in sickness observed in a comparison group exposed to the test stimulus on consecutive days without being exposed to the training environment. Similarly, the levels of sickness observed on training days 2 through 4 could be compared against sickness observed in a comparison group for which contrast and optic flow were held constant over the 3 days. These avenues were not explored for two reasons; first, these measurements were not possible because they would have required tripling the number of subjects enrolled in our study. Due to our restrictive inclusion criteria (see methods) and COVID-19 concerns it was already a challenge to recruit the current pool of subjects. Second, these methodologies were already largely exploratory, with previous literature already investigating much of the aforementioned control results. As mentioned in the related works section, it is well-known that consistent exposure to the same VR environment results in adaptation (Reason and Brand, 1975; Cobb et al., 1999; Hill and Howarth, 2000; Bailenson and Yee, 2006; Domeyer et al., 2013; Beadle et al., 2021), but these effects have also been known to dissipate in as little the same day (Lampton et al., 2000). This makes it unlikely that the results from day 5 are solely due to exposure to the city environment on the first day. Similarly, investigations into adaptation transfer have been largely unsuccessful or conflicting, so it is noteworthy that scores between days 4 and 5 were so similar. We suggest that this methodology was successful because it borrows concepts from motion sickness training such as gradually increasing exposure (Rine et al., 1999; Takeda et al., 2001) to a root cause of VR sickness (i.e., optic flow or the associated vection).

From our results, we concluded that subjects experienced lower levels of sickness over time, and these effects persisted between environments. The mechanisms behind this change, whether they be through the actions of the subjects or via some neurological mechanism, are unfortunately beyond the scope of this study. However, future work exploring changes in behavior in subjects in VR over time would be a worthy follow-up to this study.

Subjective feelings of vection were not directly measured during this experiment. However, previous research has shown increasing luminance contrast increases feelings of vection (Sauvan and Bonnet, 1993; Patterson and York, 2009; Guo et al., 2021), and so it is logical to assume the same effect was experienced in our experiment. Independent of this matter, further research has shown that increases in optic flow lead to greater feelings of VR sickness (Davis et al., 2015). Therefore, regardless of vection, we can assume that the increase in luminance contrast and optic flow did lead to greater stimulus intensity as intended.

Regarding physiological data, we used the Empatica E4 watch, which is limited to a sampling rate of 4 Hz for EDA data. It is important to note that EDA signals are particularly sensitive to motion artifacts. Because our subjects were using their hands in order to manipulate the controller for locomotion, these artifacts were likely present in our data.

## 7 CONCLUSION

In this study, we tested a novel method for managing VR sickness through training. We used an abstract environment and reduced stimulus contrast to limit sickness during training. We showed that sickness remained constant or decreased on consecutive training days, even though contrast and optic flow strength was increasing. We also argue that adaptation developed in an abstract training environment transferred to a richer test environment. These results demonstrate that adaptation to VR sickness via careful and gradual exposure to successively more provocative VR stimuli shows promise as a VR sickness mitigation strategy.

## DATA AVAILABILITY STATEMENT

The raw data supporting the conclusion of this article will be made available by the authors, without undue reservation.

## Supplementary Material

Supplemental Material

## Figures and Tables

**TABLE 1 |:** Summary results of discomfort scores, simulator sickness questionnaire, optic flow, and electrodermal data for the testing days in terms of *mean (standard deviation)*.

	Day 1	Day 5
Discomfort Score		
Average	3.08 (2.2)	1.71 (1.5)
Ending	3.84 (3.3)	2.32 (2.2)
SSQ Subscores		
Nausea	40.67 (35.7)	12.05 (24.4)
Oculomotor Discomfort	26.33 (17.2)	13.96 (21.9)
Disorientation	51.28 (38.3)	32.24 (39.1)
Total Severity	42.72 (30.8)	20.28 (27.4)
Optic flow		
Mean Magnitude	1.31 (0.4)	1.35 (0.3)
Electrodermal Data		
Ampl. RMS Baseline	0.43 (0.3)	0.44 (0.4)
Ampl. RMS End	0.51 (0.3)	0.33 (0.3)
Tonic SCL End	5.00 (5.4)	3.66 (3.5)

**TABLE 2 |:** Summary results of discomfort scores, simulator sickness questionnaire, optic flow, and electrodermal data for the training days in terms of *mean (standard deviation)*.

	Day 2	Day 3	Day 4
Discomfort Score			
Average	2.27 (2.1)	1.6 (1.5)	1.66 (1.4)
Ending	2.63 (2.4)	2.4 (2.6)	2.32 (2.2)
SSQ Subscores			
Nausea	20.59 (21.9)	19.58 (26.9)	12.55 (18.2)
Oculomotor Discomfort	23.94 (25.2)	13.96 (21.6)	17.16 (20.8)
Disorientation	37.36 (36.6)	33.70 (40.5)	33.70 (34.8)
Total Severity	29.92 (28.6)	23.62 (29.4)	22.44 (24.6)
Optic flow			
Mean Magnitude	4.83 (0.7)	7.79 (1.2)	11.57 (1.1)
Electrodermal Data			
Ampl. RMS Baseline	0.44 (0.3)	0.37 (0.4)	0.34 (0.3)
Ampl. RMS End	0.5 (0.4)	0.38 (0.4)	0.44 (0.3)
Tonic SCL End	3.65 (3.1)	4.06 (4.2)	4.01 (2.9)
