# Supplementary material for: VR Sickness Adaptation With Ramped Optic Flow Transfers From Abstract To Realistic Environments
Source: Front Virtual Real. Author manuscript; Available in PMC 2023 Mar 2. (PMC9979719; doi:10.3389/frvir.2022.848001)
Supplement: Supplemental Material [file NIHMS1868906-supplement-Supplemental_Material.pdf]

Supplementary Material

1. Do you consistently experience any stomach problems?
    a. Yes
    b. No
    c. Prefer Not to say

2. Do you have any inner ear problems?
    a. Yes
    b. No
    c. Prefer Not to say

3. Are you currently taking any medications which have a side effect of inducing nausea?
    a. Yes
    b. No
    c. Prefer Not to say

4. Do you have vertigo?
    a. Yes
    b. No
    c. Prefer Not to say

5. Do you have any vision problems (other than glasses)?
    a. Yes
    b. No
    c. Prefer Not to say

6. If you require glasses, are you willing to wear contacts for the duration of the experiment?
    a. Yes
    b. No
    c. I don't wear glasses
    d. Prefer Not to say

7. How much experience have you had with virtual reality?
    a. No Experience
    b. Very little experience (once or twice, for less than an hour total)
    c. Moderate experience (3 or more times, greater than an hour total)
    d. Very experienced (more than 5 times, for a few or more hours total)
    e. Prefer not to say
    f. Other:

8. How often do you use virtual reality (if at all)?
    a. I've never used VR
    b. Rarely (once or twice total)
    c. Somewhat infrequently (once or twice a year)
    d. Frequently (once or twice a month)
    e. Very frequently (weekly or even daily)
    f. Prefer not to say
    g. Other:

9. When I use VR I feel sick/get headaches/feel tired/etc...
    a. All the time
    b. Often
    c. Rarely
    d. Never
    e. I've never used VR (or don't know)
    f. Prefer not to say
    g. Other:

10. In terms of motion sickness in general, I would say I'm....
    a. Very sensitive to motion sickness
    b. Somewhat sensitive to motion sickness
    c. I very rarely experience motion sickness
    d. I never experience motion sickness
    e. Prefer not to say
    f. Other:

11. In your experience over the last 10 years (approximately), for each of the following types of transport or entertainment please indicate how often you felt sick or nauseated

	Not Applicable or Never Traveled	Never Felt Sick	Rarely Felt Sick	Sometimes Felt sick	Frequently felt sick
Cars					
Buses or Coaches					
Trains					
Aircraft					
Small Boats					
Ships (e.g. channel ferries)					
Roundabouts in playgrounds					
Big dippers, funfair (carnival or amusement park) rides					

Figure 1: Prescreening questionnaire provided to subjects prior to acceptance into the study. Questions 1 through 4 were taken directly from Kinsella2018AdaptationAdaptation, while questions 7 through 10 were adaptations of questions asked in the original questionnaire to allow subjects to provide more context if they have used VR or experience VR and/or motion sickness. Question 11 was taken directly from [?] with the purpose of determining if subjects are susceptible to sensory conflict or more generally, motion sickness. Subjects were excluded if they reported stomach problems, inner ear problems, medications which induce nausea, vertigo, vision problems other than glasses, or if they required glasses during the experiment and could not wear contacts. Subjects were also excluded if they responded as being "Very experienced" with VR (question 7), using VR "frequently" or more (question 8) of if they responded to questions 9 through 11 by indicating they never experience motion and VR sickness, and have never felt sick in any of the question 11 categories.
